# Clinical characteristics of epilepsy of unknown origin in the Rottweiler breed

**DOI:** 10.1186/s13028-015-0168-1

**Published:** 2015-11-06

**Authors:** Linda Heske, Izabella Baranowska Körberg, Ane Nødtvedt, Karin Hultin Jäderlund

**Affiliations:** Department of Companion Animal Clinical Sciences, Faculty of Veterinary Medicine Biosciences, Norwegian University of Life Sciences, P.B. 8146 Dep, 0033 Oslo, Norway; Department of Animal Breeding and Genetics, Swedish University of Agricultural Sciences, Box 7023, 750 07 Uppsala, Sweden; Department of Women’s and Children’s Health, Karolinska Institutet, 171 77 Stockholm, Sweden; Department of Production Animal Clinical Sciences, Faculty of Veterinary Medicine and Biosciences, Norwegian University of Life Sciences, P.B. 8146 Dep, 0033 Oslo, Norway

**Keywords:** Seizure, Dog, Rottweiler, Phenotype

## Abstract

**Background:**

Epilepsy is one of the most common neurological conditions in dogs. Despite that epilepsy appears to be common in the Rottweiler breed, published literature about the phenotype of epilepsy in this breed is lacking. The aim of this questionnaire-based study was to describe the clinical characteristics of epilepsy in the Rottweiler breed including; signalment, pedigree, housing conditions and information about the seizures such as age at onset, seizure type, duration, and progression, as well as number of seizure days (24 h), effect and side effects of anti-epileptic drugs, and potential comorbidities. The diagnosis for epilepsy of unknown origin was based on the following inclusion criteria: ≥2 seizure days, starting between 6 months and 7 years of age, no known history of poisoning or serious head trauma, and (when available), pre-study routine serum biochemical parameters were within the reference intervals.

**Results:**

A total of 37 cases (23 females and 14 males) were included in the study. The median age at onset of seizures was 36 months (range 8–84 months). The dogs suffered from generalized tonic–clonic seizures, and more than 50 % of the dogs had experienced cluster seizures (>1 seizure in 24 h). The dogs commonly started to seizure while resting (23/36) and/or sleeping (20/36). Only 3 of the 36 dogs experienced seizures during activities such as walking or training. All of the 24/37 (64.9 %) dogs on antiepileptic drugs received phenobarbital. Five dogs needed add-on treatment (n = 5), and of these: one dog was on 3 drugs (phenobarbital, potassium bromid and levetiracetam) (n = 1), three dogs were on phenobarbital and potassium bromide (n = 3), and one dog received phenobarbital and imepitoin (n = 1). Seizure frequency did not necessarily improve following antiepileptic treatment, and for six of 21 (28.6 %) of the dogs, seizure frequency increased. All of the Rottweilers in this study had relatives with epilepsy reported.

**Conclusions:**

The Rottweilers suffering from epilepsy in this study presented with generalized tonic–clonic seizures, and their response to antiepileptic treatment was variable. More than 50 % of the dogs had experienced cluster seizures (>1 seizure in 24 h).

**Electronic supplementary material:**

The online version of this article (doi:10.1186/s13028-015-0168-1) contains supplementary material, which is available to authorized users.

## Background

Epilepsy is one of the most common neurological conditions in dogs [1]. The disorder is characterized by recurrent seizures originating from the brain, due to an imbalance between excitatory and inhibitory mechanisms [2]. Epilepsy has often been divided into symptomatic epilepsy (SE), caused by an identifiable structural lesion in the brain, and idiopathic epilepsy (IE) without an underlying morphological disease. Dogs with IE frequently require lifelong antiepileptic treatment. Approximately 20–30 % of dogs with IE do not respond to antiepileptic therapy [2], and dogs with epilepsy are reported to have an increased risk of premature death, most commonly due to euthanasia, if the seizures cannot be controlled [3–7].

The prevalence of epilepsy in dogs is difficult to establish because of variable diagnostic criteria and vaguely defined populations at risk, but it was estimated to be around 1–2 % in a hospital-based referral population [8] and 0.62 % for dogs attending primary care veterinary clinics in the UK [9]. In a large cohort study of insured dogs in Sweden, the incidence rate of epilepsy (including both idiopathic and symptomatic cases) was estimated to be 18 cases per 10,000 dog-years at-risk counted as time from the first insurance date until exiting the database [10]. However, the disease prevalence is higher within breeds that are predisposed to epilepsy [11–14].

A hereditary component of IE has been described in a number of dog breeds, including Labrador Retriever, Belgian Shepherd, Boxer, Hungarian Vizsla, English Springer Spaniel, Irish Wolfhound and Border Collie [13, 15–23]. Differences in the clinical manifestations of the disease have been described both between and within breeds, and the phenotype of epilepsy in each breed should therefore be reported separately.

Our group has published results from a large cohort study of epilepsy among insured Swedish dogs and showed that the incidence rate for epilepsy in the Rottweiler breed is above average, and that the risk of premature death due to epilepsy is high for affected dogs [10]. Additionally, veterinary practitioners, as well as Rottweiler breeders and owners, in Sweden have noticed that the occurrence of epilepsy has seemed to increase in the breed over the last decades (www.rottweilerklubben.se). Epilepsy appears to be common in Rottweilers, but published literature of the phenotypic description of epilepsy in this breed is largely lacking.

The aim of this study was to describe the clinical characteristics of epilepsy in the Rottweiler breed.

## Methods

### Study population

Names, registration numbers in the Swedish or Norwegian Kennel Club (and thereby the pedigrees), and blood samples of purebred Rottweilers with epileptic seizures, living in Sweden and Norway, were collected between 2007 and 2013, in collaboration with the Swedish Rottweiler Club, the Swedish University of Agricultural Sciences and the Norwegian University of Life Sciences. The study was approved by the local Animal Ethics Committee in Uppsala (no. C138/6 and no. C139/9). The project was announced in the Swedish Veterinary Journal, the Journal of the Swedish Rottweiler Club, and at meetings for breeders and owners. Dogs were recruited from breeders and veterinary clinics all over Sweden, and to a lesser extent Norway, on a volunteer basis. Information to the owners was available in the Journal of the Swedish Rottweiler Club and online (http://hunddna.slu.se). Bio-bank consent forms (signed by the dog owners, allowing us to publish the information recorded and obtain blood samples from their dog for future research) and a questionnaire enquiring about their dog’s health could be downloaded directly from the website. Owners of dogs that had a diagnosis of epilepsy of unknown origin, and opted to participate in the study, made an appointment with their local veterinarian to have blood-samples drawn and to be included in the study.

### Questionnaire

Questionnaires were used to record detailed information about the dogs’ health (Additional file 1) and were completed by the owners when their dogs were blood sampled at the veterinary clinic. Questions about the dogs included signalment, pedigree, housing conditions and information about the seizures such as age at onset, seizure type, duration, and progression, as well as number of seizure days (24 h). Several seizures per day (24 h) were counted as one seizure day/one event), effect and side effects of anti-epileptic drugs, and potential comorbidities. It was possible to select more than one answer for many of the questions and not all owners responded to all questions. Owners also reported information about other diseases and the diagnostic work-up performed at the time of the initial diagnosis.

The diagnosis of epilepsy was based primarily on the answers to the detailed questionnaires, supplied with medical records when available. All dogs in this study met the following inclusion criteria for epilepsy of unknown origin: ≥2 seizure days, starting between 6 months and 7 years of age, no known history of poisoning or serious head trauma, and (when available), pre-study routine serum biochemical parameters were within the reference intervals. Dogs with sufficient follow-up time (≥4 months) without progression or a diagnosis of symptomatic epilepsy could be classified as suffering from IE. Some of the dogs included in this study had a follow-up time of less than 4 months; hence, not all dogs included fulfilled the criteria for IE.

### Serum samples

Serum samples were stored at −80 °C until analysed at the Central Laboratory at the Norwegian University of Life Sciences. A full serum biochemical profile was performed, including: aspartate aminotransferase (AST), alanine aminotransferase (ALT), alkaline phosphatase (ALP), creatine kinase (CK), amylase (AMY), lipase (LIP), C-reactive protein (CRP), total protein (TP), albumin (Alb), globulin (Glob), urea, creatinine (Crea), bile acids, total bilirubin (T bili), cholesterol (Chol), inorganic phosphorus, calcium (Ca), sodium (Na), potassium (K), Na/K ratio, chloride (Cl), and a thyroid profile (TT4, FT4, and TSH).

### Pedigree analysis

All but one case were purebred Rottweilers registered in the Swedish or Norwegian Kennel Clubs. The last dog was not registered, but both its parents were registered in the Swedish Kennel Club. The pedigrees of affected dogs were assessed to identify common ancestors.

## Results

### Descriptive statistics

#### Study population

A total of 37 cases (23 females and 14 males) were included in this study. All of the dogs were living indoors with their owners as family members, rather than in outdoor kennels or in separate doghouses. 16 of the dogs were active working dogs, implying that they were trained to compete in obedience, tracking, searching, or were patrol dogs in the Swedish army. Median bodyweight was 42 kg (range 31–60 kg). Ten of the dogs were neutered surgically; two additional dogs were chemically neutered. Most of the dogs were characterised by their owners as active (27), friendly (31) and calm (14) by nature. A few dogs were characterised as nervous (5), shy (1) or aggressive (3). It was possible to choose several options to describe each dog.

### Seizure database

**Fig. 1:**
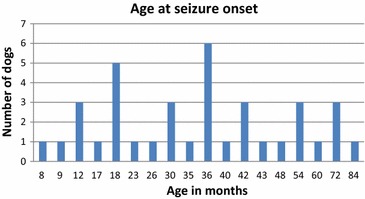
Owner-reported age at seizure onset in months, for 37 Swedish Rottweilers with epilepsy

The median age at onset of seizures was 36 months (range 8–84 months), seemingly with two peaks: At 18 and 36 months of age (Fig. 1). Twenty-three of 37 dogs (62.2 %) had their initial seizure by 36 months of age. The date of seizure onset was not reported for one dog and that dog was therefore excluded from the study. The follow-up time from the initial seizure until study inclusion varied from 1 to 60 months with a median follow-up time of 10 months. In total, 26/37 (70.3 %) of the included dogs had a follow-up time of >4 months without progression to more serious illness or SE, and these dogs fulfilled the criteria for IE. Seizure frequency ranged from several seizures per week to one per year.

Thirty-three of 35 (94.3 %) of the dogs appeared to lose consciousness during the seizures, and 26/27 (96.3 %) did not respond to contact (when the owner called their name) during the seizures. The clinical presentations of the seizures are summarized in Table 1. The dogs suffered from generalized tonic–clonic seizures with the following signs; Stiffness in neck and legs, unconsciousness, salivation, shaking, gazing, falling to the side, muscle twitching/shivering, chewing, contraction of facial muscles, and urination (whereas defecation was uncommon). The most common behaviours reported in the postictal phase were confusion and disorientation, restlessness and tiredness (Table 2).

**Table 1:** Owners’ description of behaviors observed during epileptic seizures among 37 Swedish Rottweilers

Behavior	Always	Often	Seldom	Never
Unresponsive/Unconscious	32/35 (91.3)	1/35 (2.9)	1/35 (2.9)	1/35 (2.9)
Gazing	28/33 (84.8)	2/33 (6.1)	–	3/33 (9.1)
Stiffness in neck and legs	30/36 (83.3)	4/36 (11.1)	1/36 (2.8)	1/36 (2.8)
Falling to one side	28/34 (82.3)	2/34 (5.9)	–	4/34 (11.8)
Salivating	28/36 (77.8)	3/36 (8.3)	1/36 (2.8)	4/36 (11.1)
Shaking	27/36 (75.0)	4/36 (11.1)	1/36 (2.8)	4/36 (11.1)
Muscle shivering	26/35 (74.3)	3/35 (8.6)	2/35 (5.7)	4/35 (11.4)
Contracting facial muscles	19/31 (61.3)	1/31 (3.2)	1/31 (3.2)	10/31 (32.3)
Chewing	18/34 (52.9)	4/34 (11.8)	3/34 (8.8)	9/34 (26.5)
Dilated pupils	13/28 (46.4)	3/28 (10.7)	1/28 (3.6)	11/28 (39.3)
Interacting with people	13/30 (43.3)	3/30 (10.0)	–	14/30 (46.7)
Dark in eyes	11/27 (40.7)	4/27 (14.8)	1/27 (3.7)	11/27 (40.7)
Changing body position	12/31 (38.7)	3/31 (9.7)	–	16/31 (51.6)
Anxious/fearsome	12/31 (38.7)	3/31 (9.7)	1/31 (3.2)	15/31 (48.4)
Disoriented	13/34 (38.2)	6/34 (17.7)	5/34 (14.7)	10/34 (29.4)
Turning head to one side	12/32 (37.5)	2/32 (6.2)	3/32 (9.4)	15/32 (46.9)
Urinating	10/33 (30.3)	11/33 (33.3)	4/33 (12.1)	8/33 (24.3)
Occasional apnea	9/31 (29.0)	2/31 (6.5)	3/31 (9.7)	17/31 (54.8)
Occasional blindness	8/31 (25.8)	3/31 (9.7)	1/31 (3.2)	19/31 (61.3)
Nystagmus	6/30 (20.0)	6/30 (20.0)	2/30 (6.7)	16/30 (53.3)
Barking	5/31 (16.1)	–	–	26/31 (83.9)
Aggressive	3/31 (9.7)	–	3/31 (9.7)	25/31 (80.6)
Walking in circles	2/30 (6.7)	1/30 (3.3)	3/30 (10.0)	24/30 (80.0)
Defecating	2/30 (6.7)	–	6/30 (20.0)	22/30 (73.3)
Chasing tail	–	3/30 (10.0)	2/30 (6.7)	25/30 (83.3)

The numbers of dogs by frequency of manifestation is given in n/N (%). The behaviors are ranked by decreasing frequency

**Table 2:** Owner description of how 37 Rottweilers with epilepsy mostly behave directly after seizures

Behavior	Number of dogs/total number (%)
Seem confused/disoriented	26/27 (96.3)
Restless	27/30 (90.0)
Seem to be tired	22/29 (75.9)
Drinking	18/27 (66.7)
Eating	14/27 (51.9)
Wants to go out	13/26 (50.0)
Seem to be blind	8/25 (32.0)
Unwilling to rise	6/25 (24.0)
Seem to be aggressive	4/25 (16.0)
Something else	4/30 (13.3)
Vomiting	3/24 (12.5)

The numbers of dogs are given in n/N (%). (The response rate varied and it was possible to choose several options)

Twenty of 37 (54 %) of the dogs had experienced cluster seizures (>1 seizure in 24 h). The mean number of seizure days (24 h), for the time-period included in the questionnaire, was 12.2 seizure days (range 2–67 seizure days). Only 24 owners described how the seizure frequency changed over time, (independently of antiepileptic medication): For 8/24 (33.3 %) of the dogs the frequency had decreased, for 11/24 (45.8 %) the frequency was stable, and for the remaining 5/24 (20.8 %) the frequency had increased. For 19/32 (54.9 %) of the dogs, the duration and intensity of the seizures remained the same, while it had increased for 5/32 (15.6 %), and decreased for 8/32 (25 %). The changes in seizure frequency, and intensity and duration of the seizures were based on the owners’ assessment. The dogs commonly started to seizure while resting (23/36) or sleeping (20/36). Only three of the 36 dogs (8.3 %) experienced seizures during activities such as walking or training. For the females, five of 23 bitches tended to have seizures around the time of heat; two during heat, and three within 2 months after heat. One male dog was reported to have more frequent seizures when there were females in heat in the neighbourhood.

Thirty of 37 (81.1 %) of the dogs were considered normal by the owner between seizures (the inter-ictal phase). Of the remaining dogs, five of seven were on phenobarbital and showed behavioural changes most consistent with effects of the antiepileptic medications, such as ataxia and sedation. One of the dogs on antiepileptic medications was reported to be restless and anxious in a way that it had not been previously, during the inter-ictal phase. The two dogs that were not on medication were both reported to be more apprehensive and irritated than before the seizures started.

### Diagnostic work-up in the field

Most of the dogs diagnosed with epilepsy (35/37) presented to a veterinarian because of the seizures. The diagnostic evaluation included haematology and a serum biochemical profile (32/37), and further investigations performed for some of the dogs included cerebrospinal fluid (CSF) analysis (n = 1), magnetic resonance imaging (MRI) (n = 2), computed tomography (CT) (n = 1), and abdominal ultrasound (n = 5).

### Other diseases

Six of 37 (16.2 %) dogs in this study had been treated for other diseases: sub aortic stenosis (n = 1; diagnosed as puppy and receiving treatment with a β-blocker), cranial cruciate ligament rupture (n = 3; receiving NSAIDs (nonsteroidal anti-inflammatory drugs) (for pain relief)), and hypothyroidism (n = 2; supplemented with levothyroxine). None of dogs diagnosed with epilepsy had sustained brain injuries resulting in loss of consciousness during their lifetime. None of the dogs for which information about the neonate period was available (n = 15) received intensive care or help from the breeder to survive the paediatric period.

### Antiepileptic treatment

Fourteen of the dogs were not on any antiepileptic medication at the time of data collection; because of infrequent seizures, newly diagnosed disease or the owners’ concern for potential side-effects of the antiepileptic treatment. Twenty-four dogs received phenobarbital, and five of these received add-on treatments: potassium bromide and levetiracetam (n = 1), potassium bromide (n = 3), or imepitoin (n = 1). The median time from seizure onset to initiation of antiepileptic drugs was 36 days (range: 1–730 days); 90 % of the dogs were on medication within 6 months from seizure onset. Serum phenobarbital levels were monitored in 15/24 (62.5 %) of the treated dogs. Following initiation of antiepileptic treatment, the seizure frequency decreased for 15/21 (71.4 %) of the dogs, but for 6/21 (28.6 %) the seizure frequency increased. Only 8/18 (44.4 %) dogs experienced less severe or shorter seizures after initiation of medical therapy (data was missing for remaining dogs due to short treatment time). The most common side effects of the antiepileptic drugs were polyphagia (7/21), polydipsia (7/21), exercise intolerance (7/21), reduced ability to focus (6/21), sedation (6/21), and ataxia (5/21). Reduced working ability was reported as a side effect by the owners of 8/21 (38.1 %) of the dogs. Seven of the treated dogs reported no side effects of antiepileptic medications. Only six dogs were administered additional rectal diazepam, during seizures. Three owners gave their dogs dietary supplements such as cobalamin and magnesium. Seven dogs were neutered after the onset of seizures, but none of these experienced reduced seizure frequency or intensity after the procedure. Three dogs were euthanized before the time-period of 4 months, due to severe seizures, poor seizure control and severe side effects of the anti-epileptic medication. One of these owners, with small children in the household, also reported that they were afraid of their dog’s aggressive behaviour during the post-ictal phase.

### Laboratory results

Serum from 29 dogs was analysed, for the remaining 9 cases the serum was unavailable for analysis. Seven of the dogs had increased values of urea, Crea, and Glob, and 12 of the dogs had an increased ALP activity. All remaining serum biochemical values were within the reference intervals provided by the laboratory.

### Family history and pedigree

All of the 37 dogs in this study had close relatives (siblings, parents, offspring) or second-degree relatives (grandparents, cousins) with epilepsy and 22/37 (59.5 %) of the dogs shared one common ancestor. This dog was used extensively for breeding during 1992–1997, fathering almost 300 offspring and grandfathering 850 in Sweden only.

## Discussion

This retrospective case-series provides the first detailed clinical characterisation of epilepsy of unknown origin in the Rottweiler breed.

Although the median age at seizure onset was 36 months in this study, five of the dogs were older than 5 years of age (60 months) at seizures onset (Fig. 1). Age at seizure onset in this study was similar to that reported for some breeds with IE: Italian Spinones (39 ± 19 months) [24], Hungarian Vizslas (36 months) [21] and English Springer Spaniels (36 months) [20], but higher than for Australian Shepherds (30 months), Border Collies (28.4 months) [17] and Bernese Mountain dogs (26.5 months) [25].

There was a slight majority of female dogs (1.6:1) included in this study, but because the cases were recruited based on owners volunteering, no statement can be made about sex predominance. Most studies of breed-related epilepsy show a male predominance to epilepsy, for instance in the Australian Shepherd [26], Beagle [27] Bernese Mountain dog [25], Golden Retriever [28] and Keeshond [29]. The Swedish cohort study of the incidence of canine epilepsy also showed a male predominance across breeds (male to female ratio: 1.4:1) [10].

According to the study performed by Heske et al. [10], the incidence rate of epilepsy in the Rottweiler breed was 24.3 (95 % confidence interval (CI) 19.5–29.1) per 10,000 dog-years at-risk, which is higher than the overall incidence rate of 18 (95 % CI 18–19) for all breeds. The study included IE as well as SE cases, but based on a validation study of the same database it was concluded that a majority of the cases were idiopathic [30], lending further support to the impression of a relatively high prevalence of a possible idiopathic epilepsy in this breed. Heske et al. [10] further showed that the mortality rate from epilepsy in the Rottweiler breed was 21.5 (95 % CI 16.5–26.5), which is significantly higher than the general mortality rate among epileptic dogs of all breeds of 11 (95 % CI 11–12) [10]. The survival time after a diagnosis of epilepsy was similar to other working breeds, such as Border Collies and German Shepherds, but significantly shorter than for dogs kept solely for companionship, such as Poodles, and smaller breeds like Dachshunds and Border Terriers [10]. The higher mortality rate and shorter life expectancy for Rottweilers with epilepsy compared to other breeds might be due to the characteristics of the disease, inadequate treatment or poor seizure control. Furthermore, adverse side effects of anti-epileptic treatment could be a contributing factor, and this is in agreement with the owners’ reports from the questionnaires for those dogs that were euthanized. The shorter survival time for Rottweilers and other larger breeds could also be related to management issues due to the size of the dog.

In our study, 54 % of the dogs had suffered from cluster seizures. This is similar to Australian Shepherds with IE, where 48 % of the dogs experienced cluster seizures [26], but less than in a population of Italian Spinones in the UK, where cluster seizures occurred in 85 % [24], and in a study of Border Collies with confirmed IE which revealed that cluster seizures appeared in 94 % of the cases [17].

Pharmacological resistance, or poor seizure control (defined as 1 seizure day/month despite adequate therapy with at least one anti-epileptic drug; phenobarbital with or without add-on treatment) [26], has been reported in breeds such as Border Collies [17, 31] and Australian shepherds [26]. In our study, the seizure *frequency* increased for 6/21 (28.6 %) of the dogs, and 10/18 (55.6 %) of the dogs showed no improvement in terms of seizure *duration and intensity*, following antiepileptic treatment.

Seven dogs in this study were neutered after the onset of seizures and none of them experienced reduced seizure frequency or intensity after the procedure. This is in line with Weissl et al. [26] reporting that 13 dogs were neutered after seizure onset and only one dog improved in seizure frequency and severity [26]. Results from Monteiro et al. [5] might suggest a potential therapeutic role for neutering, due to the fact that entire dogs were more likely to have cluster seizures, and with increased frequency [5], but further studies are necessary to elucidate this relationship.

The diagnosis of canine epilepsy depends heavily on historical information from the owner, including a careful description of seizure semiology. Ideally, in addition, each dog should undergo a complete diagnostic evaluation, including neurological examination, MRI, and CSF analysis, to rule out structural brain diseases causing symptomatic forms of epilepsy. The term “Epilepsy of unknown origin (EUO)” was recently introduced for a group of dogs diagnosed with epilepsy based on a history of seizures, in the absence of other medical problems determined by unremarkable clinical examination results and routine blood analyses [9]. Epileptic dogs with evidence in previous records of underlying disease that potentially could have caused epilepsy, including advanced brain imaging abnormalities, were excluded from the EUO group of dogs [9]. Due to the retrospective nature of the present study, the limited availability of specialists, and the costs of performing advanced diagnostic imaging and further investigations, the vast majority of dogs have not undergone a complete diagnostic evaluation. However, according to Armaşu et al. [32], age at seizure onset was highly predictive in differentiating dogs with EUO (mean age at seizure onset 39.8 ± 26.4 months) from epileptic dogs with structural diseases in the brain [32].

Information about epilepsy occurring in close relatives is considered important for classification as IE. All dogs included in this study had first- or second degree relatives with epilepsy. In addition, to fulfil the criterion of IE, the patients had to have an age of onset of ≥6 months and ≤7 years, and a follow-up time-period of ≥4 months after the first seizure, with no other neurological signs developing, consistent with the classification used in a previous study [30]. At least 70 % of the dogs included in this study most likely suffered from IE, as these had a follow-up time of more than 4 months. There is no reason to suspect symptomatic epilepsy in the remaining dogs, but due to the limited follow-up time, a diagnosis of IE is less certain in these patients.

Some of the serum biochemical parameters (urea, Crea, Glob and ALP) were above the reference intervals. 12 of 28 (42.9 %) dogs had increased levels of ALP. Ten of these 12 dogs received phenobarbital, likely explaining the increased ALP activity [33], the other two were barely above the reference interval. For the remaining parameters, increases may be partially explained by the relatively narrow reference intervals of our diagnostic laboratory. Using the reference intervals from the published literature (IRIS guidelines; www.iris-kidney.com) the values for 100 % of our dogs would be considered normal for Crea and Glob, and 96.6 % for Urea. Also, there is nothing in these serum biochemistry results leading us to believe that the dogs suffered from seizures of other reasons than epilepsy.

This study consists of a case-series of Swedish Rottweilers with owner-reported epilepsy. The diagnosis is validated against published diagnostic criteria [30] based on detailed questionnaires, and the majority of the included dogs are believed to suffer from IE. Because the dogs were recruited to the study based on owners volunteering to participate, the included individuals are likely to represent animals with owners that are very focused on the disease or actively associated with the breeding club. We still consider the described seizure characteristics to be representative of seizures among Swedish Rottweilers in general, although it is possible that those with poorer seizure control were not included-as owners of such dogs may be less likely to participate.

## Conclusion

Rottweilers suffering from epilepsy in this study presented with generalized tonic–clonic seizures, and their response to antiepileptic treatment was variable. More than 50 % of the dogs had experienced cluster seizures (>1 seizure in 24 h).

## Additional file

10.1186/s13028-015-0168-1 Questionnaire used in the study to record detailed information about the dogs’ health.

## Abbreviations

ALB: albumin
ALP: alkaline phosphatase
ALT: alanine aminotransferase
AMY: amylase
AST: aspartate aminotransferase
Ca: calcium
Cl: chloride
Chol: cholesterol
CI: confidence interval
CK: creatine kinase
Crea: creatinine
CRP: C-reactive protein
CSF: cerebrospinal fluid
CT: computed tomography
EUO: epilepsy of unknown origin
FT4: free T4
Glob: globulin
IE: idiopathic epilepsy
K: potassium
LIP: lipase
MRI: magnetic resonance imaging
Na: sodium
NSAIDs: nonsteroidal anti-inflammatory drugs
T bili: total bilirubin
P: total protein
TSH: thyroid stimulating hormone
TT4: total T4
SE: symptomatic epilepsy
