# Supplementary material for: Clinical characteristics of epilepsy of unknown origin in the Rottweiler breed
Source: Acta Vet Scand. 2015 Nov 6;57:75. doi: 10.1186/s13028-015-0168-1 (PMC4636809; doi:10.1186/s13028-015-0168-1)
Supplement: Supplementary file 1 — 10.1186/s13028-015-0168-1 Questionnaire used in the study to record detailed information about the dogs’ health. [file 13028_2015_168_MOESM1_ESM.pdf]

Epilepsi i ________________rasen; Hälsoformulär till djurägaren i ett forskningsprojekt med syfte att finna den genetiska bakgrunden till epilepsi i rasen.

Datum:____________________

Djurägarens namn:____

Djurägarens personnummer (frivilligt):____

Alla aktuella telefonnummer:____

E-mailadress:____

Hundens tilltalsnamn:____

Hundens namn i registreringsbeviset:____

Hundens reg.nr (alt. föräldrarnas reg.nr):____

Hundens vikt:___________________________

1. Är hunden kastrerad?
( ) ja, datum:___________________________
( ) nej

2. Är hunden en aktivt arbetande brukshund?
( ) ja, ange typ av aktivitet:____
( ) nej

3. Finns det andra djur i hemmet?
( ) ja, ange vilka:____
( ) nej

4. Hur skulle Du vilja beskriva Din hunds personlighet?
( ) Livlig
( ) Gladlynt
( ) Lugn
( ) Mindre livlig
( ) Nervös
( ) Blyg
( ) Aggressiv
( ) Annat, vad?____

5. Hur många timmar per dygn har hunden vanligtvis sällskap av någon människa, så att denne skulle kunna se eventuella epilepsianfall med egna ögon?
( ) Mindre än 5 timmar per dygn
( ) 5-10 timmar per dygn
( ) 10-15 timmar per dygn
( ) 15-20 timmar per dygn
( ) mer än 20 timmar per dygn
6. Bor hunden vanligtvis inomhus eller utomhus?____________________________________

Allmänna frågor runt Din hunds epilepsi:
7. När/i vilken ålder hade hunden sitt första epileptiska anfall?_________________________

En del hundar med epilepsi har fler än ett anfall under samma dygn när de väl har sina anfall, s k clusterepilepsi.
8. Har Din hund haft mer än ett anfall under samma dygn någon gång?
( ) ja
( ) nej (hoppa till fråga 13 direkt)

9. Hur många anfall hade hunden totalt under det dygn den hade sitt första anfall?__________

10. Hur många anfall under ett och samma dygn har hunden haft som mest?______________

11. Hur många anfall under ett och samma dygn har hunden haft som minst?______________

12. Hur många anfall under ett och samma dygn har Din hund vanligtvis?________________

13. När hade hunden sitt/sina senaste epilepsianfall?__________________________________

14. Vid (ungefär) hur många tillfällen (hur många olika dygn) har hunden haft epileptiska anfall hittills i livet?____

15. Hur ofta kom det ett dygn med epilepsianfall i början av sjukdomstiden?
( ) var_______ dag
( ) var _______vecka
( ) var_______ månad
( ) var_______ år

16. Hur ofta kommer det ett dygn med epilepsianfall nuförtiden?
( ) var_______ dag
( ) var _______vecka
( ) var_______ månad
( ) var_______ år

SLU

UPPSALA UNIVERSITET

17. Hur har längden och intensiteten på epilepsianfallen utvecklats med tiden?
( ) det har minskat påtagligt
( ) det har minskat något
( ) det har fortsatt vara likadant
( ) det har ökat något
( ) det har ökat mycket

18. Hur lång tid gick det från det första epilepsianfallet tills hunden sattes på epilepsimedicin?
( ) hunden står inte på någon kontinuerlig epilepsimedicinering
( )_______dagar
( )_______veckor
( )_______månader
( )_______år

19. Finns det något mönster i när hundens epilepsianfall uppträder?
( ) under stress
( ) en speciell tid under dygnet, nämligen____
( ) när den varvat ner efter att ha varit mycket stressad
( ) under löpningstid (gäller tikar)
( ) under de närmaste två månaderna efter löpningen (gäller tikar)
( ) när tikar i grannskapet löper (gäller hanhundar)
( ) något annat mönster, nämligen____
( ) det går inte att identifiera något mönster

20. Om Din hund kastrerades när den hade haft epilepsi ett tag, hur påverkades epilepsin av det?
( ) det blev betydligt glesare mellan anfallstillfällena
( ) det blev något glesare mellan anfallstillfällena
( ) det ändrades inte alls
( ) det blev anfall ännu tätare efter kastrationen

21. Förutom den tid det tar precis efter ett anfall innan hunden blir ”sig själv” igen, beter sig Din hund helt normalt mellan anfallstillfällena?
( ) ja
( ) nej; Vad är annorlunda nu mot förr?____

22. Har Din hund nära släktingar med epilepsi?
( ) ja
( ) jag vet inte
( ) nej
Ange här vilka släktingar med epilepsi Du känner till (gärna med reg.nr och/eller namn i reg.beviset):____
____
____

SLU

UPPSALA UNIVERSITET

Frågor runt tiden strax innan Din hund får ett epileptiskt anfall:
23. I vilken/vilka situationer får Din hund vanligtvis sina anfall?
( ) i vila
( ) under sömn
( ) vaken, i normal aktivitet
( ) under hög fysisk aktivitet
( ) under mental stress
( ) när hunden saknar Dig (eller annan familjemedlem)
( ) efter utfodring
( ) när hunden är som mest hungrig
( ) när hunden är sjuk på något annat sätt
( ) när hunden har någon intensiv känsloupplevelse (t ex när den visar aggressivitet, eller är i slagsmål)
( ) epilepsianfallen kommer inte i någon speciell situation och utan att vara förknippade med någon speciell känsloupplevelse

24. Kan Du märka innan ett anfall att det ska komma?
( ) ja
( ) nej (hoppa direkt till fråga 28)
25. På vilket sätt märks det att hunden skall få ett anfall?
( ) illamående
( ) kräkning
( ) salivering, dregling
( ) rastlöshet
( ) hunden söker kontakt med ägaren
( ) hunden blir aggressiv
( ) det märks på något annat sätt, vilket?____
____
____
____

26. Hur lång tid innan anfallet märker Du att det skall komma?
( ) mindre än 1 minut
( ) 1-5 minuter
( ) 5- 30 minuter
( ) 30-60 minuter
( ) 1-2 timmar
( ) 2-6 timmar
( ) 6-12 timmar
( ) 12-24 timmar
( ) 1-2 dagar
( ) mer än 2 dagar

27. Hur ofta kan Du förutsäga att Din hund ska få ett anfall?
( ) aldrig
( ) 25% av gångerna
( ) 50% av gångerna
( ) 75% av gångerna
( ) alltid

Frågor runt själva epilepsianfallet:
28. Har Du någonsin sett Din hund ha ett epilepsianfall?
( ) ja ( ) nej

29. Har Du någonsin sett ett helt anfall, från början till slut?
( ) ja ( ) nej

30. Vad gör Din hund precis före ett anfall?
( ) sover
( ) är vaken
( ) går på promenad ute
( ) leker
( ) är med ägaren på träning/sport
( ) något annat, vilket?____
____

31. Kan Du beskriva i detalj vad som sker precis innan anfallet börjar?__________________
____
____

32. Har Du någonsin försökt få Din hund att reagera på sitt namn, eller få kontakt med den på något annat sätt, medan hunden har sitt anfall?
( ) ja
( ) nej (hoppa till fråga 34)

33. Om Du svarade ja på förra frågan, beskriv hur hunden reagerade:
( ) helt normalt (fullt kontaktbar)
( ) onormalt, men inte helt okontaktbar (reagerade på tal eller att man rörde vid den)
( ) helt okontaktbar (reagerade inte på något sätt på tal eller att man rörde vid den)

34. Ungefär hur lång tid håller själva kramperna på under ett enstaka epilepsianfall?
Vanligtvis håller krampanfallet på i ungefär _________ minuter
Det kortaste anfallet höll på i ungefär __________minuter
Det längsta anfallet höll på i ungefär _________ minuter

Beskrivning av epilepsianfallet:

35. Uppskatta hur typiska följande alternativ är för hundens anfall. (Snälla, svara på alla rader.) I rutan som står före respektive alternativ, är det meningen att Du skall numrera så att det framgår i vilken ordning de olika symtomen uppträder under anfallet. Om mer än ett symtom uppträder samtidigt, kan Du skriva samma siffra på dem.

( ) Stelnar i nacke och ben	( ) alltid	( ) ofta	( ) sällan	( ) aldrig
( ) Ramlar omkull	( ) alltid	( ) ofta	( ) sällan	( ) aldrig
( ) Muskeldarrningar	( ) alltid	( ) ofta	( ) sällan	( ) aldrig
( ) Skakningar	( ) alltid	( ) ofta	( ) sällan	( ) aldrig
( ) Vrider huvudet åt ena sidan	( ) alltid	( ) ofta	( ) sällan	( ) aldrig
( ) Drar ihop ansiktsmusklerna	( ) alltid	( ) ofta	( ) sällan	( ) aldrig
( ) Urinerar	( ) alltid	( ) ofta	( ) sällan	( ) aldrig
( ) Har avföring	( ) alltid	( ) ofta	( ) sällan	( ) aldrig
( ) Tillfälligt andningsuppehåll	( ) alltid	( ) ofta	( ) sällan	( ) aldrig
( ) Dreglar	( ) alltid	( ) ofta	( ) sällan	( ) aldrig
( ) Tuggar	( ) alltid	( ) ofta	( ) sällan	( ) aldrig
( ) Ändrar kroppsställning	( ) alltid	( ) ofta	( ) sällan	( ) aldrig
( ) Jagar sin svans	( ) alltid	( ) ofta	( ) sällan	( ) aldrig
( ) Går i cirkel	( ) alltid	( ) ofta	( ) sällan	( ) aldrig
( ) Tillfälligt okontaktbar	( ) alltid	( ) ofta	( ) sällan	( ) aldrig
( ) Pupillerna är vidgade	( ) alltid	( ) ofta	( ) sällan	( ) aldrig
( ) Svartnar i ögonen	( ) alltid	( ) ofta	( ) sällan	( ) aldrig
( ) Stirrande blick	( ) alltid	( ) ofta	( ) sällan	( ) aldrig
( ) Flackande blick (nystagmus)	( ) alltid	( ) ofta	( ) sällan	( ) aldrig
( ) Söker sig till folk	( ) alltid	( ) ofta	( ) sällan	( ) aldrig
( ) Går in i möbler o dyl	( ) alltid	( ) ofta	( ) sällan	( ) aldrig
( ) Tillfälligt blind	( ) alltid	( ) ofta	( ) sällan	( ) aldrig
( ) Skäller	( ) alltid	( ) ofta	( ) sällan	( ) aldrig
( ) Visar rädsla	( ) alltid	( ) ofta	( ) sällan	( ) aldrig
( ) Visar aggressivitet	( ) alltid	( ) ofta	( ) sällan	( ) aldrig

36. Har alla Din hunds anfall varit likadana?
( ) ja
( ) nej. På vilket sätt har de varit olika?____

____

37. Har Du någonsin fått ett intryck av att ena kroppshalvan, eller någon annan del av hundens kropp, uppför sig annorlunda än resten av kroppen under ett anfall? Till exempel krampar mer intensivt etc.
( ) ja; Hur?____
( ) nej

38. Har Du någonsin kunnat påverka hur ett anfall förlöper?
( ) ja; Hur?____
( ) nej

39. Tror Du att Din hund efteråt är medveten om att den har haft ett epileptiskt anfall?
( ) ja
( ) nej
Varför tror Du det?____
____

<u>Nu följer ett antal frågor runt hur Din hund beter sig strax efter det epileptiska anfallet, under den sk post-iktala fasen, som kan vara från någon minut till flera timmar till i enstaka fall några dygn efter ett epileptiskt anfall.</u>

40. Är Du rädd för Din hunds reaktioner efter ett anfall?
( ) ja
( ) nej
Varför?____
____

41. Reagerar Din hund när Du kallar den vid namn direkt efter ett krampanfall?
( ) ja ( ) nej

42. Har Du någonsin försökt få Din hund att lyda ett kommando direkt efter ett krampanfall?
( ) ja ( ) nej

43. Om Du svarade ja på förra frågan, vad hände?
( ) Hunden utförde kommandot på ett normalt sätt
( ) Hunden uppfattade kommandot, men utförde inte uppgiften på det sätt han normalt gör
( ) Hunden lydde inte alls

Beskrivning av förloppet efter själva epilepsianfallet:
44. Hur lång tid tar det efter ett anfall innan hunden är sig själv igen?
( ) mindre än 5 minuter
( ) 5-15 minuter
( ) 15-30 minuter
( ) 30-60 minuter
( ) 1-2 timmar
( ) 2-6 timmar
( ) mer än 6 timmar, nämligen ca ______ timmar
( ) hunden beter sig helt normalt direkt efter anfallet

UPPSALA UNIVERSITET

45. Uppskatta hur typiska följande alternativ är för tiden närmast efter hundens anfall. I rutan som står före respektive alternativ, är det meningen att Du skall numrera så att det framgår i vilken ordning de olika symtomen uppträder efter anfallet. Om mer än ett symtom uppträder samtidigt, kan Du skriva samma siffra på dem.

( ) Hunden är trött	( ) alltid	( ) ofta	( ) sällan	( ) aldrig
( ) Vandrar omkring	( ) alltid	( ) ofta	( ) sällan	( ) aldrig
( ) Visar aggressivitet	( ) alltid	( ) ofta	( ) sällan	( ) aldrig
( ) Dricker	( ) alltid	( ) ofta	( ) sällan	( ) aldrig
( ) Äter	( ) alltid	( ) ofta	( ) sällan	( ) aldrig
( ) Vill gå ut	( ) alltid	( ) ofta	( ) sällan	( ) aldrig
( ) Vill inte kliva upp	( ) alltid	( ) ofta	( ) sällan	( ) aldrig
( ) Kräks eller har kväljningar	( ) alltid	( ) ofta	( ) sällan	( ) aldrig
( ) Verkar blind	( ) alltid	( ) ofta	( ) sällan	( ) aldrig
( ) Beter sig förvirrat	( ) alltid	( ) ofta	( ) sällan	( ) aldrig

( ) Något annat, vad?____

____

____

Hundens hälsa och veterinärundersökningar:

46. Har en veterinär undersökt Din hund pga epilepsianfallen?

( ) ja ( ) nej

47. Gjordes några ytterligare undersökningar med anledning av det?

Blodprovsundersökningar	( ) ja	( ) nej
Ryggmärgsvätskeprov (CSF)	( ) ja	( ) nej
Magnetkameraundersökning (MR)	( ) ja	( ) nej
Datortomografiundersökning (CT)	( ) ja	( ) nej
Elektroencephalografi (EEG)	( ) ja	( ) nej
Något annat	( ) ja	( ) nej

Om ja, vad?____

48. Vilka veterinärer/kliniker har Du besökt med Din hund pga epilepsianfallen?__________

____

49. Har Din hund, vid sidan av epilepsi, några andra hälsoproblem av betydelse?

( ) ja, vad?____

( ) nej

50. Står Din hund på någon långvarig medicinering, förutom epilepsimediciner?

( ) ja, vad?____

( ) nej

51. Har Din hund tidigare i livet varit med om någon skallskada som resulterade i tillfällig medvetslöshet och/eller andra symtom på hjärnskada?
( ) ja, när?____
( ) nej

52. Har Din hund tidigare i livet någonsin diagnosticerats med hjärnhinneinflammation (meningit) och/eller hjärninflammation (encephalit)?
( ) ja, när?____
( ) nej

53. Känner Du till något om hur det var när hunden föddes?
( ) ja
( ) nej (hoppa direkt till fråga 57 eller 60)

54. Vilken var hundens födelsevikt?____________________________________

55. Var det några problem runt förlossningen?
( ) ja, vad då?____
( ) nej

56. Behövde hunden som nyfödd valp (under de första veckorna i livet) någon hjälp av människor för att överleva?
( ) ja, vad då?____
( ) nej

Frågor som endast gäller tikar:
57. Hur gammal var Din hund när hon löpte första gången?_______________________

58. Är löpningarna regelbundna? (Om tiken är kastrerad; Var löpningarna regelbundna före kastrationen?)
( ) ja ( ) nej

59. Har Din hund fått valpar?
( ) ja, hur många kullar?____
( ) nej

Frågor som endast gäller hanhundar:
60. Har Din hund ett normalt sexualbeteende?
( ) ja
( ) nej; Hur är det onormalt?____
____
____

61. Har Din hund avkommor?
( ) ja, hur många kullar?____
( ) nej

Epilepsimedicinering:

62. Får din hund någon/några mediciner mot sin epilepsi?
( ) ja
( ) nej (hoppa till fråga 72)

63. När påbörjades medicineringen?____

64. Vilken/vilka epilepsimediciner får Din hund regelbundet?

Medicin 1, namn:____
Antal mg per tablett, eller per ml:_____________________________________
Antal tabletter, eller ml, per doseringstillfälle:_________________________
Antal gånger per dygn som hunden medicineras:_____________________

Medicin 2, namn:____
Antal mg per tablett, eller per ml:_____________________________________
Antal tabletter, eller ml, per doseringstillfälle:_________________________
Antal gånger per dygn som hunden medicineras:_____________________

Medicin 3, namn:____
Antal mg per tablett, eller per ml:_____________________________________
Antal tabletter, eller ml, per doseringstillfälle:_________________________
Antal gånger per dygn som hunden medicineras:_____________________

65. Får hunden sin medicin kontinuerligt?
( ) ja
( ) nej. När (och varför just då) får den sin medicin?________________________________
____

66. Har medicinnivåerna i blodet mätts på Din hund?
( ) ja, resultat (om Du vet):____
____
( ) jag vet inte
( ) nej

67. Hur effektivt kontrolleras epilepsianfallen av medicineringen?
( ) anfallen har helt upphört
( ) anfallen har glesnat till ungefär hälften så ofta eller ännu glesare
( ) anfallen har glesnat, men bara lite
( ) anfallen kommer lika ofta som eller oftare än innan medicineringen påbörjades

68. Har anfallen blivit lindrigare eller kortare efter det att hunden sattes på medicinering?
( ) ja, hur?____
( ) nej

69. Har Du märkt av några biverkningar från medicineringen (förutom de biverkningar som vanligtvis är tydliga under den första behandlingsveckan)?
( ) Jag har inte märkt några biverkningar
( ) Sömnighet
( ) Kräkningar
( ) Ökad aptit
( ) Ökad törst
( ) Vinglighet, ostadighet
( ) Minskat tålamod
( ) Sämre uthållighet
( ) Sämre koncentrationsförmåga
( ) Mera lättstressad
( ) Något annat, vad?____

70. Har Din hunds förmåga att ”jobba” påverkats av medicineringen?
( ) ja, hur?____
____
( ) nej

71. Ger Du någon extra medicin till hunden just när den har anfall?
( ) ja. Vilken medicin och vilken dos?____
( ) nej

72. Har Din hund fått någon annan slags behandling (tex naturmedicin, kosttillskott, akupunktur) för sin epilepsi?
( ) ja,
( ) nej (hoppa till punkt 76)

73. Vilken/vilka andra behandlingar har Din hund fått? ________________________________
____
____

74. Hur länge har hunden fått denna/dessa behandlingar?_______________________________
____

75. Hur effektivt kontrolleras epilepsianfallen av denna/dessa behandlingar?______________
____
____
____

76. Har Du någon övrig information, som Du tror kan vara värdefull för oss att få veta, får Du gärna skriva det här:

Tusen tack för att Du bidrar till denna viktiga forskning! Med Din hjälp hoppas vi kunna förstå oss på sjukdomen epilepsi bättre i framtiden, och därigenom förbättra möjligheterna att både förebygga och behandla denna diagnos.

Det ifyllda hälsoformuläret skickas, tillsammans med biobanksmedgivande och ev. journalkopior, till:

"Namn på projekt"/Tomas Bergström
Husdjursgenetiska Laboratoriet
Sveriges Lantbruksuniversitet
Box 7023
750 07 Uppsala
